# Analysis of some common pathogens and their drug resistance to antibiotics

**DOI:** 10.12669/pjms.291.2744

**Published:** 2013

**Authors:** Lidao Bao, Rui Peng, Xianhua Ren, Ruilian Ma, Junping Li, Yi Wang

**Affiliations:** 1 Lidao Bao, Department of Pharmacy, Affiliated Hospital of Inner Mongolia Medical University, Hohhot-010059, People’s Republic of China.; 2 Rui Peng, Department of Pharmacy, Affiliated Hospital of Inner Mongolia Medical University, Hohhot-010059, People’s Republic of China.; 3 Xianhua Ren, Department of Pharmacy, Affiliated Hospital of Inner Mongolia Medical University, Hohhot-010059, People’s Republic of China.; 4 Ruilian Ma, Department of Pharmacy, Affiliated Hospital of Inner Mongolia Medical University, Hohhot-010059, People’s Republic of China.; 5 Junping Li, Department of Pharmacy, Affiliated Hospital of Inner Mongolia Medical University, Hohhot-010059, People’s Republic of China.; 6 Yi Wang, Department of Pharmacy, Affiliated Hospital of Inner Mongolia Medical University, Hohhot-010059, People’s Republic of China.

**Keywords:** Antibacterial drugs, Pathogen, Gram-negative bacteria, Bacteria resistance

## Abstract

***Objective:*** To investigate the common bacterial resistance of clinical isolates in our hospital in the second half of 2011.

***Methodology:*** Pathogens isolated from clinical samples in the second half of 2011 were analyzed and categorized to perform susceptibility tests.

***Results:*** In the gram-negative bacteria, *Enterobacteriaceae* and non-fermenting gram-negative bacilli accounted for 55.89% and 34.51%. In the gram-positive bacteria, *Staphylococcus aureus*, *Coagulase-negative staphylococci*, *Enterococcus*, *Strptococcus pneumonia* accounted for 32.85%, 40.39%, 12.41% and 10.22%, respectively. Other species accounted for 4.14%. *Klebsiella pneumonia* and *Pseudomonas aeruginosa* were sensitive to cepoperazon, cefepime and imipenem. However,*Acinetobacter baumannii* was more sensitive to carbapenems antibiotics, which was followed by fourth generation cephalosporins. *Klebsiella pneumoniae* was extremely sensitive to amikacin, cefepime and imipenem, but was resistant to ampicillin. The detection rates of the broad-spectrum *Escherichia coli*, Pseudomonasaeruginosa and *Klebsiella pneumoniae* were 54.51%, 52.08% and 38.65%. The gram negative bacilli were the prevalent clinical pathogens in our hospital in the second half of 2011.

***Conclusion:*** The drug resistance of pathogenic bacteria has increased significantly recently, thus the surveillance of antibacterial agents is necessary, and rational use of antibiotic will be urgently needed to reduce the production and dissemination of drug resistant strains.

## Introduction

 Bacterial resistance has become one of the most important issues in the global medical field. It differs in various regions and is related with many social factors.^1^ Analysis of the resistance can assist to observe the characteristics and changes of the bacterial resistance. Besides, it can provide real-time reference for the clinical application of antibiotics and enhance the monitoring of the distribution of common bacteria, which can offer epidemiology data and changes of bacterial resistance for clinic, and effectively guide the clinical treatment for pathogen infections.^2^^,^^3^ The analysis of the isolated bacterial resistant in our hospital in the second half of 2011 is presented in this manuscript.

## Methodology

***Source of bacterial strains: ***All pathogens were isolated from community-acquired infections of the patients enrolled in our hospital, and the secondary infections after being enrolled were excluded.

***Instruments and reagents: ***Automatic micro-analyzer, ProtoCOL automated microbial analysis system, bacterial identification cards and drug susceptibility test cards^4^ were bought from Synbiosis (UK) Ltd. and control strains including *Staphylococcus aureus* ATCC25923, *Klebsiella pneumoniae* ATCC700603, *Escherichia coli *ATCC25922, *Pseudomonas aeruginosa* ATCC27853, *Candida albicans* ATCC90029 and *Enterococcus faecium*ATCC33186 were all purchased from Shanghai Chuan Xiang Biotech Co., Ltd.

***Drug sensitivity tests:*** Bacterial identifications and susceptibility tests were performed through Automatic micro-analyzer (British Synbiosis) and ProtoCOL automated microbial analysis system, respectively. Antimicrobial minimum inhibitory concentration (MIC) was carried out by the automatic analyzer. Drug sensitivity tests met the standards of Clinical and Laboratory Standards Institute (CLSI).^5^

***Assay methods:*** The disc confirmatory tests of ESBLs (extended spectrum β lactamases) of *Escherichia coli*, *Klebsiella pneumonia* and *Proteus mirabilis* were carried out according to CLSI2008. Quality control strains included *Escherichia coli* ATCC25922 (negative control) and *Klebsiella pneumoniae* ATCC700603 (positive control). PRSP (penicillin resistant *Streptococcus pneumoniae*) were detected by Optoching antibacterial tests and *Streptococcus pneumoniae* monoclonal latex agglutination tests. Drug sensitive test of *Streptococcus pneumoniae* was carried out by ATB-STREP5-test paper, and *Streptococcus pneumoniae* were divided into three categories: penicillin-susceptible strains (PSSP), penicillin-intermediate strains (PISP) and penicillin-resistant strains (PRSP).^6^

***Statistical analysis:*** The results were analyzed by one-way ANOVA for multiple comparisons by SPSS. A P-value < 0.05 was considered significant.

## Results

 A total of 1096 isolates were collected in the second half of 2011, the sources of the strains included respiratory secretions (68.80%), urine (15.69%), blood (12.14%) and others (3.38%).

 In the 1096 isolates, gram negative and gram positive bacteria accounted for 54.20% and 37.50% respectively, fungi accounted for 8.30%. In the gram negative bacteria, the proportion of *Enterobacteriaceae* was 55.89%, and those of non-fermenting gram negative bacilli and other species were 34.51% and 9.60%, respectively. In the gram positive bacteria, *Staphylococcus*, *Coagulase-negative staphylococci*, *Enterococcus* and *Streptococcus pneumonia* accounted for 32.85%, 40.39%, 12.41% and 10.22% respectively, other species accounted for 4.14%. The proportions of all the bacteria are shown in Table-I. The main pathogens, including *E. coli*, *Staphylococcus aureus*, *Klebsiella pneumoniae*, *Pseudomonas aeruginosa*, *Candida albicans* and *Acinetobacter baumannii*, occurred following a decreasing frequency.

**Table-I:** Distribution of 1096 strains of pathogenic bacteria

*Pathogen*	*No. of strains*	*Proportion (%)*
Gram negative bacteria	594	54.20
Enterobacteriaceae	332	30.29
E. coli	153	13.96
Klebsiella pneumoniae	92	8.39
Others	87	7.94
NFGNB	205	18.70
Pseudomonas aeruginosa	74	6.75
Acinetobacter baumannii	62	5.66
Others	69	6.30
Others	57	5.20
Gram positive bacteria	411	37.50
Staphylococcus aureus	135	12.14
Coagulase-negative staphylococci	166	15.23
Staphylococcus epidermidis	52	4.74
Staphylococcus haemolyticus	51	4.65
Others	63	5.75
Enterococcus	51	4.65
Pneumonia hammer	42	3.83
Others	17	1.55
Fungi	91	8.30
Candida albicans	66	6.02
Candida glabrata	18	1.64
Others	7	0.64

***Resistance of gram negative bacilli to antibiotics:*** Top five of the selected gram negative bacilli were *Klebsiella pneumoniae*, *Pseudomonas aeruginosa*, *Acinetobacter baumannii*, *Escherichia coli* and *Proteus mirabilis*, respectively. Susceptibility tests showed that *Klebsiella pneumoniae* was sensitive to amikacin, cefepime and imipenem (the resistance rate <20%), whereas it was highly resistant to ampicillin. The detection rates of ESBLs from *Escherchia coli*, *Pseudomonas aeruginosa* and *Klebsiella pneumoniae* were 54.51%, 52.08% and 38.65%, respectively. *Pseudomonas aeruginosa* was sensitive to cefepime and imipenem, whereas it exhibited high resistance to cefoperazone/tazobactam and cefotaxime. *Acinetobacter baumannii* as well as *Escherichia coli* were resistant to most antibiotics (Table-II).

**Table-II:** Resistance rates of gram-negative bacteria to antibiotics (%).

*Antibiotic*	*Klebsiella pneumoniae (n=92)*	*Bacillus pyocyaneus (n=74)*	*Acinetobacter baumannii (n=62)*	*Escherichia coli (n=153)*	*Proteus mirabilis* *(n=32)*
Amikacin	19.57	31.08	48.39	23.53	25.00
Ampicillin	94.57	91.89	96.78	90.20	90.63
Aztreonam	61.96	85.90	67.74	71.90	34.38
Ceftazidime	34.78	33.78	79.03	70.59	40.63
Cefotaxime	33.70	29.73	75.81	57.52	43.75
Cefoperazone/ Tazobactam	27.17	28.38	70.97	50.98	31.25
Cefepime	19.57	18.92	38.71	33.99	21.88
Imipenem	10.87	12.16	24.19	28.76	15.63
Gentamicin	29.35	28.38	62.90	66.67	46.88
Ciprofloxacin	26.09	24.32	64.74	64.71	40.63
Levofloxacin	21.74	25.68	72.58	73.20	34.38

***Resistance of gram-positive cocci to antibiotics:*** Gram positive cocci mainly consisted of *Staphylococcus aureus*, *Staphylococcus epidermidis* and *Enterococcus*. *Staphylococcus aureus* was sensitive to fusidic acid, vancomycin and teicoplanin, but showed high resistant rates to β-lactams, macrolides, quinolones and aminoglycosides. *Staphylococcus epidermidis* was highly resistant to a variety of antibiotics, and *Enterococci*was resistant to traditional antibiotics. Susceptibility tests showed the detection rates of MRSA and MRCNS were 60.13% and 85.54%, in which 38 and 39 strains were resistant to fusidic acid and vancomycin, respectively (Table-III).

**Table-III:** Resistance rates of gram-positive bacteria to antibiotics (%).

*Antibiotic*	*Staphylococcus aureus (n=92)*	*Staphylococcus epidermidis (n=74)*	*Enterococcus (n=62)*	*Streptococcus pneumoniae (n=153)*	*Staphylococcus haemolyticus (n=32)*
Penicillin	95.65	95.95	96.77	96.73	93.75
Cefazolin	92.39	94.60	80.65	88.24	84.38
Gentamicin	93.48	93.24	77.42	86.27	81.25
Levofloxacin	83.70	83.78	72.58	78.43	71.88
Azithromycin	91.30	94.60	82.26	86.93	90.63
Ciprofloxacin	86.96	91.89	95.16	90.20	87.50
Cefotaxime Fusidic Acid	81.52	85.16	75.81	75.82	78.13
	41.30	31.08	35.48	50.98	50.00
Vancomycin	42.39	35.14	43.55	46.35	53.13
Teicoplanin	35.87	33.78	38.71	42.48	59.38

## Discussion

 The emergences of NDM-1 (New Delhi-Metallo-1) bacteria worldwide that are resistant to most antibiotics have greatly threatened human health.^7^ In recent years, many antibiotics are becoming invalid confronting multi-resistant bacteria. In China, the occurrences of MRSA, MDRSP and VRE have increased tremendously and covered most of the medical institutions, even *Klebsiella pneumoniae* (KPC) has also been observed having the same tendency. In future, more super bacteria resisting to most antibiotics like NDM-1 bacteria will emerge.^8^^,^^9^ Therefore, as a primary hospital, it is necessary to be aware of the distribution of common pathogens, monitor the bacterial resistance and analyze the bacterial resistance to common antibiotics.^10^

 The results showed that the resistances of *Klebsiella pneumoniae* to cefoperazone/tazobactam, cefepime, imipenem, ciprofloxacin, gentamicin and levofloxacin were not very significant. Therefore, according to the principle that antibiotics should be used following a rising sensitivity order, leveofloxacin, ciprofloxacin and cefoperazone/tazobactam could be utilized first as empirical treatment against *Klebaiella*. *Pseudomonas aeruginosa* was also sensitive to the above antibiotics, but the degree was lower than that of *Klebsiella*. The drug resistance mechanism of *Pseudomonas aeruginosa* is quite complex, which will produce a variety of β-lactamase enzymes and express active efflux system.^11^ Besides, the decreased permeability of the membrane resulted from the changes of outer membrane protein and penicillin-binding protein contributes to the resistance.^12^

 Therefore, the starting point of antibiotics for *Pseudomonas aeruginosa* ought to be higher than that for *Klebsiella*. *Acinetobacter baumannii* ranked second after *Pseudomonas aeruginosa* for the infections induced by non-fermenting gram-negative bacteria. *Acinetobacter* carried various drug resistance genes, which would bring about high risks to nosocomial infection.^13^ In the presence of the extensively used antibiotics, treating the infections resulted from multi-drug resistant *Acinetobacter baumannii* is becoming difficult. According to the bacterial resistance in the second half of 2011 in our hospital, *Acinetobacter baumannii* was sensitive to carbapenem antibiotics followed by second-generation cephalosporins, but it was highly resistant to other antibiotics, which should arouse the attention of primary health care institutions. *Enterobacteriaceae* bacteria were sensitive to carbapenem antibiotics, fourth generation cephalosporins and glycopeptides. The susceptibility tests showed that the sensitivity rates of *E. coli* to imipenem and cefepime were both above 70%, whereas it was highly resistant to other antibiotics. However, it is worthy to note that the resistance rates against *E. coli* in other medical institutions were generally lower than those in our hospital. ESBLs tests showed that the detection rate of ESBLs in *E. coli* was 54.51%, which may be related to the wide application of third generation cephalosporins, leading to the utilization of CTM-type ESBL produced by *E. coli* as the superior strain.^14^

 During the second half of 2011, the detection rates of MRSA and MRCNS were 60.13% and 85.54%, respectively. Both the bacteria were resistant to all of the β-lactam antibiotics, and they exhibited an increasing resistance tendency to fluoroquinolones, aminoglycosides and polypeptide antibiotics. In the past year, vancomycin-resistant *Staphylococcus aureus* emerged in succession and became resistant to fusidic acid and cefepime. Therefore, it has been demonstrated that the resistance to polypeptide antibiotic was severe.^15^^,^^16^

 Furthermore, *Staphylococcus epidermidis* is one of the most common *Coagulase-negative staphylococci*, which can also be isolated from most of the methicillin-resistant *Coagulase staphylococcus*, and its resistance rate increases yearly.^17^ The results exhibited that except for fusidic acid and polypeptide antibiotics, the resistance of *Staphylococcus epidermidis* to other antibiotics was even higher than that of MRSA, the severity of which should not be ignored as a result. As an opportunistic pathogen, *Enterococcus* led to increasing infections every year, and its drug resistance has become extremely serious according to the results in our hospital. Vancomycin-resistant *Enterococci *was also observed in our hospital, so it is imperative to enhance the monitoring of the drug resistance. *Streptococcus pneumoniae* is the major pathogen resulting in community-acquired infections, and its resistance to antibiotics has also aroused wide concern.^18^ Many respiratory tract infections are triggered by *Streptococcus pneumonia*. Nevertheless, in the presence of the penicillin-resistant *Streptococcus pneumoniae*, long-term macrolide antibiotics have to be utilized for the treatment, which enables the *Streptococcus pneumoniae* to be resistant to macrolide antibiotics increasingly.^19^^,^^20^ The results herein showed that the resistance rates of both azithromycin and quinolones were as high as that of penicillin for *Streptococcus pneumoniae*. Besides, the resistance values of *Streptococcus pneumoniae* to β-lactams, quinolones and macrolides antibiotics exceeded the national average levels.^21^

 In summary, it is to reinforce the surveillance of pathogens and their drug resistance to use antibiotics rationally. Clinically, antibiotics should be used according to the results of the susceptibility tests to reduce the prevalence of drug resistant strains, which will allow us to control the increase and spread of bacterial resistance.

## References

[1] Kottapalli KR, Sarla N, Kikuchi S (2006). In silico insight into two rice chromosomal regions associated with submergence tolerance and resistance to bacterial leaf blight and gall midge. Biotechnol Adv.

[2] Velickovic-Radovanovic R, Petrovic J, Kocic B, Antic S, Mitic R (2012). Analysis of antibiotic utilization and bacterial resistance changes in a surgical clinic of Clinical Centre, Nis. J Clin Pharm Ther.

[3] Xie W, Yu K, Pauls KP, Navabi A (2012). Application of Image Analysis in Studies of Quantitative Disease Resistance: Exemplified Using Common Bacterial Blight-Common Bean Pathosystem. Phytopathology.

[4] Gagliotti C, Sarti M, Sabia C, Gargiulo R, Rossolini GM, Carillo C (2011). Accuracy of automated and manual systems for susceptibility testing of Pseudomonas aeruginosa to piperacillin and piperacillin-tazobactam. New Microbiol.

[5] Taylor CR (2011). New revised Clinical and Laboratory Standards Institute Guidelines for Immunohistochemistry and Immunocytochemistry. Appl Immunohistochem Mol Morphol.

[6] Reinert RR, Al-Lahham A, Lutticken R (2001). In vitro activity of cefditoren against clinical isolates of penicillin-susceptible and penicillin-intermediate strains of Streptococcus pneumoniae isolated in Germany, 1992-1998. J Antimicrob Chemother.

[7] Walsh TR, Weeks J, Livermore DM, Toleman MA (2011). Dissemination of NDM-1 positive bacteria in the New Delhi environment and its implications for human health: an environmental point prevalence study. Lancet Infect Dis.

[8] Liu QZ, Wu Q, Zhang YB, Liu MN, Hu FP, Xu XG (2010). Prevalence of clinical methicillin-resistant Staphylococcus aureus (MRSA) with high-level mupirocin resistance in Shanghai and Wenzhou, China. Int J Antimicrob Agents.

[9] Meng X, Dong M, Wang DI, He J, Yang C, Zhu L (2011). Antimicrobial susceptibility patterns of clinical isolates of gram-negative bacteria obtained from intensive care units in a tertiary hospital in Beijing, China. J Chemother.

[10] Chiu SK, Lin JC, Wang NC, Peng MY, Chang FY (2008). Impact of underlying diseases on the clinical characteristics and outcome of primary pyomyositis. J Microbiol Immunol Infect.

[11] Walsh TR (2008). Clinically significant carbapenemases: an update. Curr Opin Infect Dis.

[12] O’Daniel PI, Zajicek J, Zhang W, Shi Q, Fisher JF, Mobashery S (2010). Elucidation of the structure of the membrane anchor of penicillin-binding protein 5 of Escherichia coli. J Am Chem Soc.

[13] Mezzatesta ML, D’Andrea MM, Migliavacca R, Giani T, Gona F, Nucleo E (2012). Epidemiological characterization and distribution of carbapenem-resistant Acinetobacter baumannii clinical isolates in Italy. Clin Microbiol Infect.

[14] Wang A, Yang Y, Lu Q, Wang Y, Chen Y, Deng L (2008). Occurrence of qnr-positive clinical isolates in Klebsiella pneumoniae producing ESBL or AmpC-type beta-lactamase from five pediatric hospitals in China. FEMS Microbiol Lett.

[15] Thong KL, Junnie J, Liew FY, Yusof MY, Hanifah YA (2009). Antibiograms and molecular subtypes of methicillin-resistant Staphylococcus aureus in local teaching hospital, Malaysia. J Microbiol Biotechnol.

[16] Zhanel GG, Adam HJ, Low DE, Blondeau J, Decorby M, Karlowsky JA (2011). Antimicrobial susceptibility of 15,644 pathogens from Canadian hospitals: results of the CANWARD 2007-2009 study. Diagn Microbiol Infect Dis.

[17] Makki AR, Sharma S, Duggirala A, Prashanth K, Garg P, Das T (2011). Phenotypic and genotypic characterization of coagulase negative staphylococci (CoNS) other than Staphylococcus epidermidis isolated from ocular infections. Invest Ophthalmol Vis Sci.

[18] Paradisi F, Corti G, Cinelli R (2001). Streptococcus pneumoniae as an agent of nosocomial infection: treatment in the era of penicillin-resistant strains. Clin Microbiol Infect.

[19] Diaz-Brito V, Gomes MH, Figueiredo-Dias P, Mota-Miranda A (2007). Leukemoid reaction and fever after polyvalent polysaccharide pneumococcal vaccination. Acta Med Port.

[20] Tremolieres F (2006). Current epidemiology of microbial low respiratory tract infections. Med Mal Infect.

[21] Njunda AL, Assob JC, Nsagha DS, Kamga HL, Awafung MP, Weledji EP (2012). Epidemiological, clinical features and susceptibility pattern of shigellosis in the Buea Health District, Cameroon. BMC Res Notes.

